# Atypical exanthem with acral involvement in adult patients associated with human herpesvirus 7 active replication: A case series

**DOI:** 10.3389/fmed.2023.1144856

**Published:** 2023-04-14

**Authors:** Andrea Michelerio, Adi Tchich, Camilla Vassallo, Valeria Brazzelli

**Affiliations:** ^1^Department of Clinical-Surgical, Diagnostic and Pediatric Sciences, University of Pavia, Pavia, Italy; ^2^Dermatology Clinic, Fondazione IRCCS Policlinico San Matteo, Pavia, Italy

**Keywords:** exanthem, atypical exanthem, skin rash, urgent care, human herpesvirus 7

## Abstract

An “atypical exanthem” (AE) is an eruptive skin eruption that differs in morphology and etiology from classical exanthems and is often a reason for urgent medical evaluation. The most frequent cause of AEs is a viral infection, but an accurate etiology cannot be established basing on the sole clinical features. Human herpesviruses (HHV) have been often suspected as etiologic agents or cofactors in atypical rashes. We performed a retrospective analysis of adult patients presenting an atypical exanthem associated with HHV-7 active replication in our center. The charts of patients were reviewed and the demographic, clinical and laboratory data collected. Nine patients (six males and three females) were included in the study, with a mean age of 43 years for men and of 26 years for women. All patients presented active HHV-7 replication in plasma during the rash, which turned negative after the exanthem resolved. The exanthem displayed a maculopapular pattern involving the trunk, limbs and, notably, the acral regions, in six patients. In three cases the exanthem was confined to only the acral sites. In most cases, there was no fever and the inflammatory indices remained unchanged. Antihistamines, topical and systemic corticosteroids were used as treatment, with excellent symptom control. We propose adding skin manifestation associated with HHV-7 to the concept of atypical exanthems, in particular those localized to the acral regions.

## Introduction

An “atypical exanthem” (AE) is defined as an eruptive skin eruption that differs in morphology and etiology from classical exanthems such as measles, scarlet fever, rubella, erythema infectiosum, exanthem subitum, and chickenpox (1). AEs may be preceded or associated with nonspecific systemic symptoms, including low-grade fever, malaise, or upper respiratory or gastrointestinal tract symptoms (1, 2). Some patients may also have an accompanying mucous membrane involvement, the “enanthem,” characterized by oral petechiae, papules, vesicles or ulcers (2). The sudden onset and extensive cutaneous involvement of AEs often cause the patient to require urgent medical evaluation.

The most frequent cause of AEs is a viral infection, followed by drug reactions, and bacteria/parasite infections (1), but an accurate etiology cannot be established basing on the sole clinical features. Yet, a correct diagnosis could be crucial for the patient and for the community, especially in case of an infectious agent (3).

Human herpesviruses (HHV) are double-stranded DNA viruses which play a definite role in a number of dermatological diseases, including genital herpes, chickenpox, shingles, Kaposi’s sarcoma, etc., but they have been suspected of causing or contributing to skin rashes as well (1, 4). However, clearly established associations are rare (5) and investigations into an etiologic association between HHVs and cutaneous diseases are complicated by their ubiquity. Human herpes virus 7 (HHV-7) is one of the eight known HHVs and was first isolated in 1990 (6). Generally the infection is asymptomatic, occurs during childhood and consequently more than 95% of adults are seropositive (7). However, despite the high prevalence, reports on the role of HHV-7 in dermatologic diseases apart from pityriasis rosea and exanthema subitum are scarce (8).

## Materials and methods

We performed a retrospective analysis of nine adult patients presenting an atypical exanthem associated with active HHV-7 replication in our third-level referral center in Lombardy, Italy, between the years 2016 and 2019.

The patients were evaluated with careful medical history, including recent travel history and drug assumption, and a complete physical examination. Extensive laboratory analyses, including routine hematochemicals, microbiological investigations, and throat swabs were performed on admission.

Microbiological investigations include anti-streptolysin-O titer, VDRL, TPHA, and IgG and IgM to Mycoplasma pneumoniae, Epstein Barr virus (EBV), Cytomegalovirus (CMV), Varicella Zoster virus (VZV), human immunodeficiency virus (HIV), Parvovirus B19, Coxsackievirus. Calibrated quantitative real-time PCR was used to evaluate presence and copy number of CMV, EBV, Human herpes virus 6 (HHV-6), HHV-7, Parvovirus B19, Coxsackievirus. If a finding was suggestive for a specific etiology, the test was repeated in the convalescence phase. A skin biopsy for histopathological examination and direct immunofluorescence was performed in doubtful cases.

The exanthem was considered associated with active HHV-7 replication if viral DNA copies could be identified in plasma using RT-PCR during the acute phase and subsided in the convalescence phase, and all other causes of exanthem were ruled out. We did not include cases of pityriasis rosea according to the criteria of Chuh et al. (9) The charts of all patients enrolled were reviewed and the demographic, clinical and laboratory data collected. This study was conducted in accordance with the principles of the Declaration of Helsinki and all patients provided informed written consent.

## Results

Nine adult patients (six males and three females) were included in the study, with a mean age of 43 years for males and of 26 years for females. The clinical data of our patients are listed in Table 1. The case of patient 1 was studied and published by the group of Brazzelli et al. (10).

**Table 1:** Clinical data of the patients.

Patient	Sex, age (years)	Lesion morphology	Lesion distribution	Itch	Duration (days)	Laboratory exams	DNA copies/ml	Treatment
1^*^	M, 50	Maculopapular	Trunk, limbs, acral skin	Intense	29	Mild lymphocytosis	800	Antihistamines and topical steroids
2	M, 53	Maculopapular, vesicles on hands	Trunk, limbs, acral skin	Intense	26	Elevated CRP	720	Antihistamines and systemic corticosteroids
3	M, 51	Maculopapular	Trunk, limbs, acral skin	Mild	16	Mild lymphocytosis, elevated CRP and ESR	29,000	Antihistamines, systemic and topical corticosteroids
4	F, 30	Maculopapular	Trunk, limbs, acral skin	Intense	22	Normal	5,220	Topical corticosteroids
5	M, 32	Maculopapular	Trunk, limbs, acral skin	Intense	10	Normal	180	Antihistamines, topical corticosteroids
6	M, 20	Maculopapular	Trunk, limbs, acral skin	Mild	24	Normal	450	Topical corticosteroids
7	F, 23	Maculopapular, vesicles on hands and feet	Acral skin	Mild	20	Elevated CRP	1,300	Topical corticosteroids
8	M, 50	Maculopapular, blisters on feet	Acral skin	None	32	Normal	1,350	Topical corticosteroids
9	F, 25	Maculopapular	Acral skin	None	20	Mild lymphocytosis	2,250	Topical corticosteroids

* Case from Brazzelli V, Giorgini C, Barruscotti S, Croci GA, Borroni G. Human herpesvirus-7 popular rash in a healthy adult patient. *Acta Derm Venereol*. 2017;97(4):537–538. doi: 10.2340/00015555.

M, male; F, female; CRP, C-reactive protein; ESR, erythrocyte sedimentation rate.

**Figure 1:**
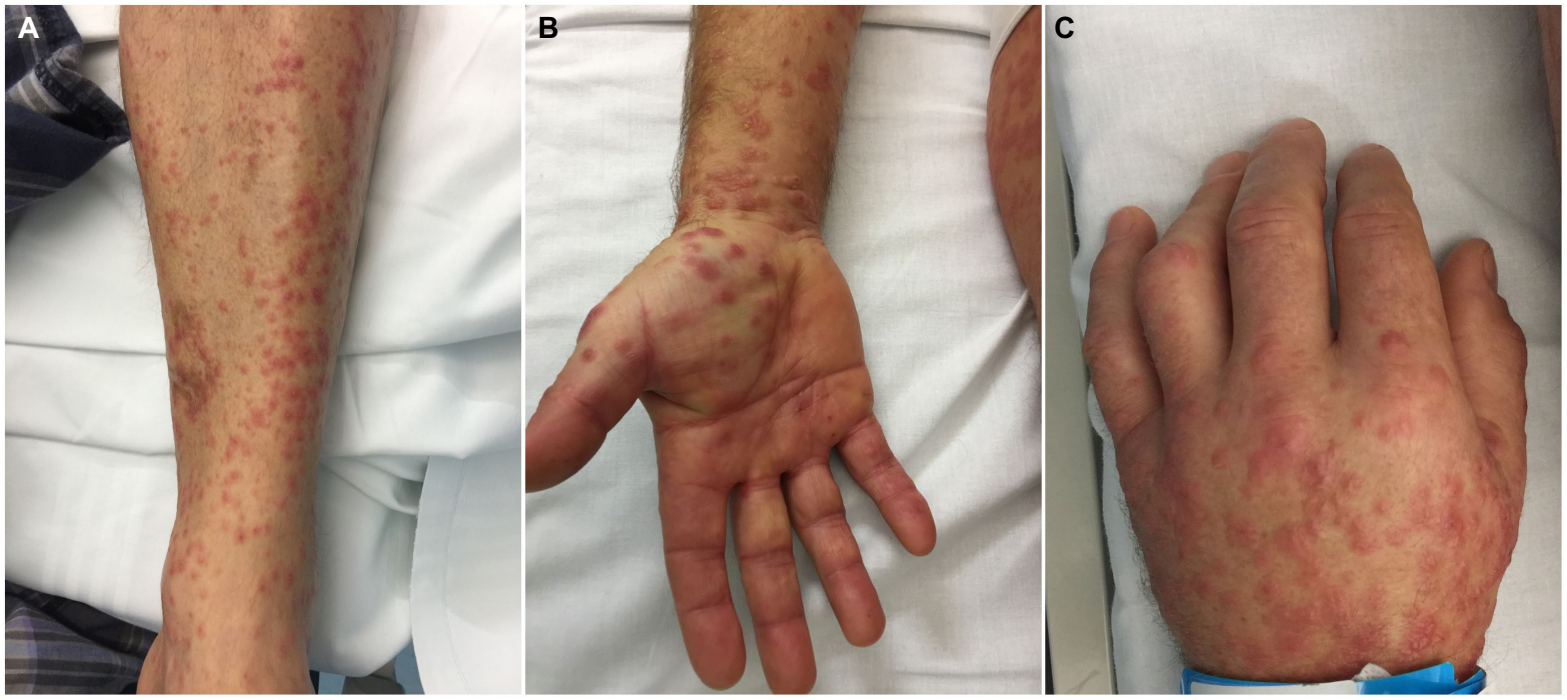
Patient 2. Erythematous maculopapular exanthem involving the limbs **(A)** and acral sites **(B,C)**. Small vesicles can be observed on the hand dorsum **(C)**.

**Figure 2:**
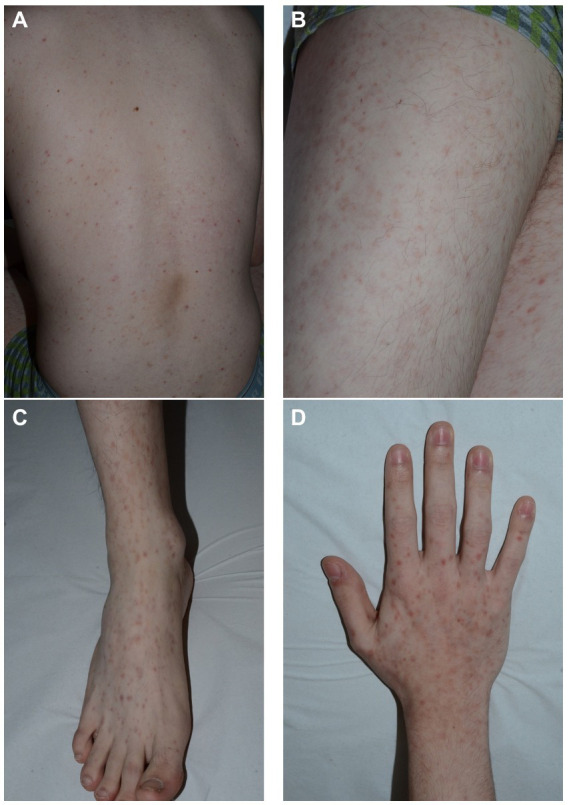
Patient 6. Erythematous maculopapular exanthem involving the trunk **(A)**, the limbs **(B)** and the acral sites **(C,D)**.

**Figure 3:**
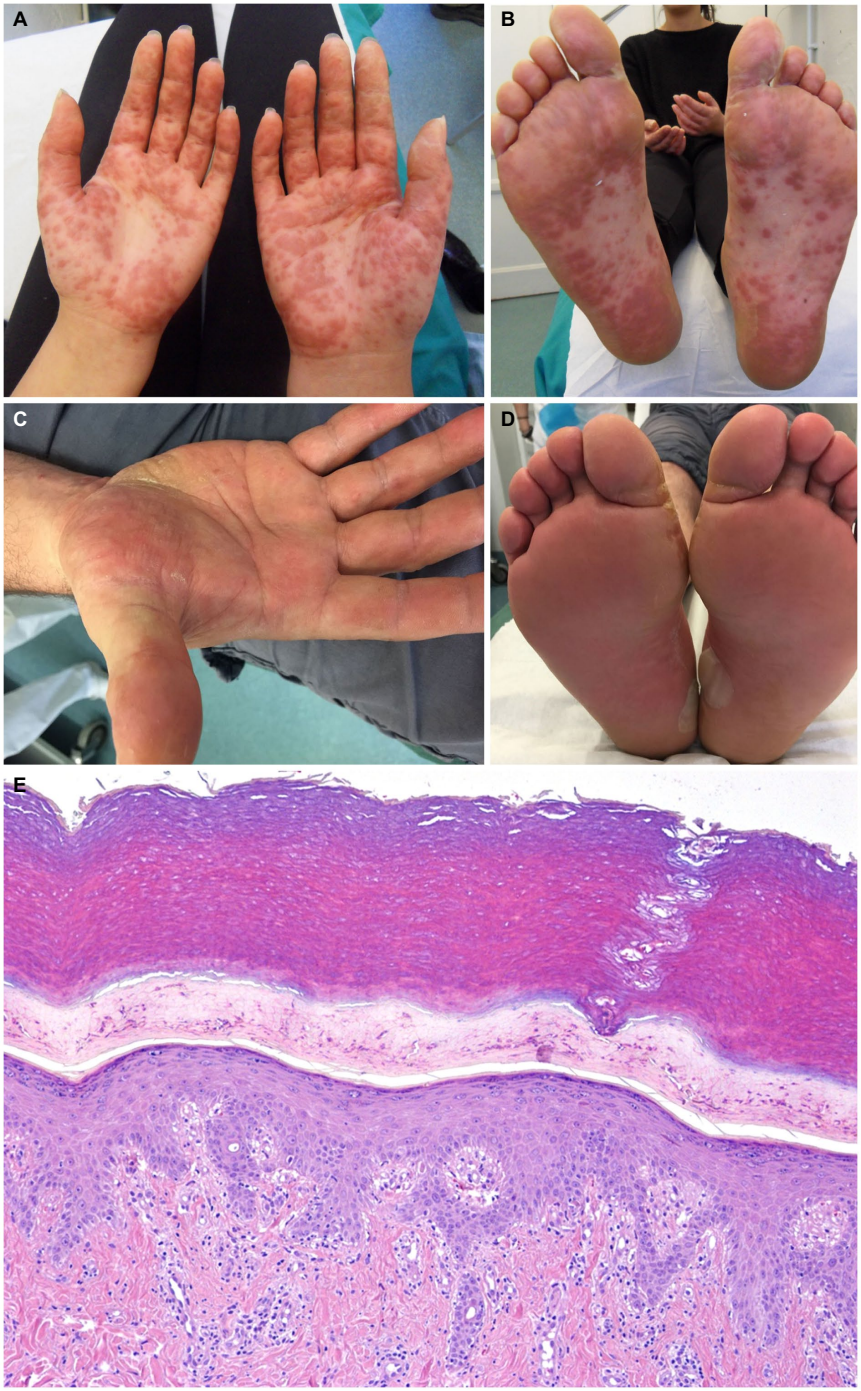
Patient 7 **(A,B)** and Patient 8 **(C,D)**. Erythematous maculopapular eruption involving the palms and the soles. Blisters are evident on the soles of patient 8 **(D)**. The histopathological sample from patient 7 shows epidermal spongiosis and an interstitial and perivascular lymphocytic infiltrate in the dermis (hematoxylin-eosin, x40) **(E)**.

**Figure 4:**
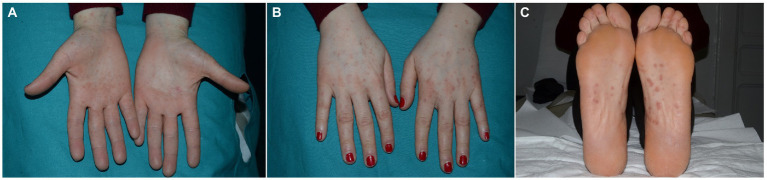
Erythematous maculopapular eruption limited to the palmar **(A-B)** and plantar **(C)** regions.

The exanthem was characterized by maculopapular erythematous lesions diffused on the trunk, limbs and acral regions in six patients (#1, #2, #3, #4, #5, and #6; Figures 1, 2), and localized only to acral sites in three patients (#7, #8, and #9; Figures 3, 4).

Three patients also had blisters and vesicles on erythematous acral skin (#2, #7, and #8; Figure 3). Despite the acral involvement, no patient developed acral edema. Ocular involvement and enanthem were not observed. Itch was present in seven patients. Two patients had fever (#3, #7).

The level of plasma viremia for HHV-7 ranged between 180 and 29,000 DNA copies/ml (median value 1,300 DNA copies/ml).

The C-reactive protein level was slightly elevated in three patients (#2, #3, #7; mean value 2.12 mg/dl, normal value 0.00–0.50) and one of them (#3) presented raised erythrocyte sedimentation rate, mild lymphocytosis, elevated liver enzymes, arthralgia and headache. Mild lymphocytosis was present in other two patients (#1, #9). One patient (#2) was hospitalized because of the extensive cutaneous involvement.

Comorbidities in our patients included diabetes mellitus type 1, diabetes mellitus type 2 and celiac disease; none was immunosuppressed.

Seven patients developed the skin exanthem in the spring and summer months, two patients in autumn. Family members did not report similar symptoms in any of the cases.

The histopathological exam was available for four patients, revealing an epidermal mild spongiosis, lymphocyte exocytosis and a superficial, perivascular and interstitial infiltrate composed mainly by lymphocytes (Figure 3D). Direct immunofluorescence studies were negative.

Antihistamines, topical and systemic corticosteroids were used as treatment, with excellent symptom control. The cutaneous lesions resolved over a period of 1 month in all cases (median duration 22 days). All patients had negative HHV-7 viral DNA after the exanthem resolved.

## Discussion

An exanthem is defined as any eruptive skin rash that may be associated with lesions of the mucous membranes (enanthem), fever or systemic symptoms. Onset and evolution of a rash often guide the clinical diagnosis. Beside the classic infectious exanthems occurring in childhood and easily recognized clinically, other exanthems with different morphology and caused by different infectious and/or toxic agents may occur. The latter are known as “atypical exanthems” and their atypic morphology, sudden onset, and extensive cutaneous involvement lead patients to seek urgent medical attention, making them one of the most common reasons for dermatological consultations.

Infectious agents are a common cause of AE, with viral infections being the most prevalent (1). The exact cause of AE can, however, be difficult to determine solely from the patient’s clinical picture, even though a precise diagnosis can be important both for the patient and for public health, especially when an infectious agent is involved.

Thus, our patients with AE underwent extensive laboratory analyses to identify an etiological agent, which resulted in HHV-7 DNA detection in plasma, a marker of active viral replication (11). Within 4 weeks from onset, all patients experienced resolution of the rash; by this time, HHV-7 DNA in blood had turned negative suggesting an association between HHV-7 replication and the exanthem. Seasonality is further evidence that a viral infection may be associated with the rash (1).

Commonly spread by saliva, HHV-7 causes a lifelong subclinical infection that is generally asymptomatic and highly prevalent within the population. Nevertheless, HHV-7 infection can cause different clinical manifestations in children, including exanthema subitum, pityriasis rosea, measles-, rubeola-like eruptions accompanied by fever, febrile seizures, and febrile status epilepticus (5, 8). HHV-7 can also contribute to the development of symptomatic CMV infections in transplanted patients, as well as serious neurologic disorders such as acute myelitis, optic neuritis, meningitis, and encephalitis (8). In immunocompetent adults, HHV-7 infection has been associated with drug reaction with eosinophilia and systemic symptoms (DRESS) and lichen planus (8), but only once with AE (10).

In our adult and immunocompetent patients, the exanthem displayed a maculopapular pattern involving the trunk, limbs and, notably, the acral regions. In the majority of cases, there was no fever and the inflammatory indices remained unchanged or just slightly elevated. Only one patient had a more severe inflammatory reaction with a mononucleosis-like syndrome, despite mild itching and a shorter than average duration of the rash. One patient, however, required hospitalization due to extensive skin involvement. Interestingly, this patient had a viral load that was lower than that of the other patients, suggesting that the cutaneous lesions may have occurred as a result of an interaction between the virus and the immune system and not to a direct cytopathic effect, as suggested by Drago et al. (12) This concept has also emerged during pandemics, where typical and atypical presentations of exanthems have been potentially linked to COVID-19 infection or vaccination (13, 14).

It is noteworthy that in some cases the exanthem was confined to only the acral sites. Indeed, the presentation of these localized cases could remember a popular purpuric “Gloves-and-Socks” syndrome (PPGSS), a self-limited rare dermatosis characterized by edema, erythema, and pruritic petechiae and papules of the dorsal and palmar surfaces of the distal extremities. This is often accompanied by systemic symptoms, including fever, lymphadenopathy, asthenia, myalgia, and arthralgias (15). Traditionally considered a distinctive manifestation of parvovirus B19 infection, PPGSS may indeed represent a nonspecific manifestation of several viral infections, including CMV, EBV, measles virus, coxsackie B6 virus and, noteworthy, human herpesvirus 6 (16, 17). In one case, a simultaneous infection of both HHV-7 and parvovirus B19 was found to cause PPGSS, indicating that multiple concurrent infections and genetic factors may be involved in its development (18). Our case is unique in that it is the first reported where parvovirus B19 was not identified, and instead, the only identified virus was HHV-7. This finding suggests that exclusive infection with HHV-7 may be sufficient for the induction of PPGSS.

Exanthems uncommonly involve the acral skin, so clinicians can narrow their differential diagnoses. Among these are infectious diseases such as meningococcal infection, secondary syphilis, Mediterranean spotted fever, hand-foot-and-mouth disease, scabies, and noninfectious conditions like erythema multiforme, Kawasaki disease, dyshidrosiform bullous pemphigoid and others (19, 20). Atypical pityriasis rosea is also a differential diagnosis. However, the acral involvement is unusual and the eruption typically presents features similar to classic pityriasis rosea, such as collarette scaling and trunk involvement (21).

The use of antihistamines, as well as topical and systemic corticosteroids, has proven to be effective in controlling symptoms in our patients. Antivirals have not been administered to any of our patients. On this matter, it should be noted that while high doses of acyclovir may have an anti-HHV-6 effect, it is unlikely to have an effect on HHV-7 (21). Foscarnet and cidofovir have shown greater effectiveness in treating HHV-7, but their use is often burdened by serious side effects such as myelosuppression and nephrotoxicity (22). Therefore, their use in self-resolving AEs may not be justified.

In conclusion, we propose adding skin manifestation associated with HHV-7 to the concept of AEs, in particular those localized to the acral regions. For a physician this means looking for new associations of diseases, new diagnostic techniques and treatments, as well as answering patients’ questions about why a particular skin disease has developed. Moreover, considering the rare but possible potentially serious consequences of HHV-7 infections (8, 23), laboratory testing for HHV-7 might be useful for AEs, especially if they involve the acral areas. In addition to being in the patient’s interest, this is also in the interest of the community in regard to time off from school and complications for pregnant women, and immunosuppressed and/or hospitalized patients.

## Limitations

This study has two main limitations. As our laboratory does not perform IgM and IgG assays against HHV-7, we cannot determine whether our patients had a first infection or a reactivation. However, the scarce sensitivity and specificity of these tests, due to cross-reactivity with other Herpesviridae such as HHV-6, hinders any solid interpretation of HHV-7 serology (4, 11). A histopathological examination was not available for all patients, as most of these patients were evaluated in emergency setting and usually re-evaluated after 1 month, when the rash had resolved.

## Data availability statement

The original contributions presented in the study are included in the article/Supplementary material, further inquiries can be directed to the corresponding author.

## Ethics statement

Ethical review and approval was not required for the study on human participants in accordance with the local legislation and institutional requirements. The patients/participants provided their written informed consent to participate in this study.

## Author contributions

All authors listed have made a substantial, direct, and intellectual contribution to the work and approved it for publication.

## Funding

Open access funding provided by Ricerca Corrente Ministero della Salute - Fondazione IRCCS Policlinico San Matteo.

## Conflict of interest

The authors declare that the research was conducted in the absence of any commercial or financial relationships that could be construed as a potential conflict of interest.

## Publisher’s note

All claims expressed in this article are solely those of the authors and do not necessarily represent those of their affiliated organizations, or those of the publisher, the editors and the reviewers. Any product that may be evaluated in this article, or claim that may be made by its manufacturer, is not guaranteed or endorsed by the publisher.
